# Challenges faced in Latin America for the implementation of an ideal health-care model for rheumatoid arthritis patients: are we ready?

**DOI:** 10.1007/s10067-015-3034-7

**Published:** 2015-08-09

**Authors:** Juan Carlos Rodríguez Jaillier, Ana María Posada Arango, David Antonio Martínez Pérez

**Affiliations:** Universidad Pontificia Bolivariana, Medellín, Colombia; Universidad Jorge Tadeo Lozano, Bogotá, Colombia; ONCOSALUD, Lima, Peru; Universidad CES, Medellín, Antioquia Colombia; Fundación Universitaria San Martin, Sabaneta, Antioquia Colombia

**Keywords:** Attention levels, Catastrophic disease, Clinical trials, Diagnostic tests, Disability, Epidemiology, Health system, Medical education, Population characteristics, Public health, Rheumatoid arthritis

## Abstract

Rheumatoid arthritis (RA) is a chronic, inflammatory, progressive disease characterized by inflammation of the synovial tissue. It results in the severe functional deterioration of the joints involved and the incapacity to work. Our main aim is to determine the characteristics of the current health-care models used in treating rheumatoid arthritis patients in Latin America. We want to analyze the details, using them as the foundation to create an ideal health-care model that is focused on the patient. We have revised documents, including guides to clinical practice, monitoring models and health-care models according to the current policies and resources available in various Latin American countries. Based on this information, the qualities and deficiencies of the current models will be analyzed, in order to use this as a basis on which to construct a proposed health-care model that covers the specific needs of rheumatoid arthritis patients, considering the resources of each population. Despite the collapse seen in many health systems throughout history, we can learn from them and should develop a new model starting from the path pursued, capitalizing on our experiences, teachings, and errors committed. However, in most cases, the obstacles to the success of the systems do not lie in the fundamental structure or the “spirit of the legislator” but rather in the day-to-day development within the community and the special interest of each agent in a system.

## Introduction

Rheumatoid arthritis (RA) is a chronic, inflammatory, progressive disease characterized by inflammation of the synovial tissue. It results in the severe functional deterioration of the joints involved and the incapacity to work. It is the most frequently seen inflammatory rheumatic disease and can involve other organs, aside from joints, including the lungs, heart, eyes, skin, and vessels. Its diagnosis is eminently clinical [1].

If a good therapeutic result is not obtained, it causes major progressive musculoskeletal disability. It is estimated that RA patients die between 8 and 12 years earlier than the general population; the primary cause of death in these patients is cardiovascular disease and arteriosclerotic events in 40 %. The cost of its impact on public health is comparable with that of heart disease [2].

The quality of life related to health (evaluated by DAS28) in RA patients is poor and comparable with that of patients suffering from chronic diseases. Rheumatic diseases are of high impact and thus require close attention. The differences mainly lie, as compared with other chronic diseases such as diabetes mellitus and chronic kidney disease, in the perception of pain and disability, which is greater in rheumatic disease groups [3].

Recent literature reveals that an early, and sometimes aggressive, approach to the pathology can significantly alter the clinical progression of the disease. Unfortunately, the health-care model in Latin American countries is based on the classification of institutions according to levels of complexity (generally classifying from level I to level III or IV, with I being the least complex and III or IV, the most specialized). This typical classification of services fragments the health-care model, ignoring the dynamics that arise in a model that is focused on the patient and their disease. In turn, this limits the appropriate attention and correct treatment by specialists during early stages of disease [4].

In the current model, following an external consultation or induced-demand consultation, most patients are directed towards promotion and prevention programs. At this point, however, the patient requires studies and specialized rheumatology care in order to be suitably classified, diagnosed, and treated. As a product of the current health systems, barriers begin to appear along the long journey before finally benefiting from treatment that is capable of changing the course of the disease [5].

A model therefore appears where the patient is the focus, which should be introduced and applied to Latin American health systems for the treatment of patients with chronic diseases. Disease management (DM) can be defined as a process to optimize public services by coordinating resources throughout the health system, during the entire disease life cycle. It is based on scientific evidence and aims to improve quality and results (clinical, economic, quality of life, and satisfaction of users and professionals) at the lowest possible cost. The essential aim of disease management programs is to limit the long-term costs on the health system. A fundamental part of this new public service philosophy is the “revolution” of the scale of priorities and values between medical professionals. A move is made away from a system that is based and focused on solving acute pathologies and problems and towards a system based on the prevention and management of chronic diseases and conditions. It heads away from a system where professionals and managers and their interests and corporate limitations play a key role, towards one in which it is the patients and the organization of their treatment that take the lead [6].

## Barriers preventing access to health care in the current health-care model

### Prohibitive costs

RA has direct, indirect, and intangible costs. Direct costs include medical and paramedical attention, treatment and diagnostic procedures, hospitalization, early retirement, etc. [5].

Indirect costs include loss of productivity and reduced income. Intangible costs include all those relating to the deterioration of patient quality-of-life [5, 7].

It should be noted that the main element of the direct medical costs of RA are the costs of the drugs or medicines that, together with scientific progress, have reduced mortality, morbidity, and disability considerably, yet the secondary cost to the use of new medications has risen [7, 8].

For example, in Colombia, the monthly cost of patient care comes to approximately US$ 1000 of which medications account for approximately 87.9 % of the relative net cost. This ends up generating higher costs for caring for patients with cardiovascular disease and a great many other entities typically considered as high-cost diseases [5].

In Mexico, it is estimated that RA has a prevalence of 1.6 % and mainly affects the age group with the greatest capacity to work and produce. This is therefore then reflected in high levels of working disability and disability pensions, which have an equally high impact on the economy. It is estimated that the direct medical cost in dollars of RA in Mexico is US$ 2334, and the cost from the patient’s own pocket is US$ 610. Reports have revealed that 15 % of the family income goes towards RA expenses, which can be disastrous on the economy of any family [9].

In Mexico, the annual cost of the care for a patient with RA is US$ 5534 (65 % direct costs and 35 % indirect costs). It has been seen that in 46.9 % of RA patients, the costs from the disease to be covered generate expenses against the household income. Together with a retrospective analysis, mainly considering the type of insurance cover offered and the duration of the disease, results in the impoverishment of 66.8 % of households, associated with catastrophic costs. The cost of RA in Mexican homes and particularly in those without full health coverage results in catastrophic costs and impoverishment [10].

### Inadequate consideration of the “high cost”

In Colombia, which today has 96 % of the population covered by insurance, a definition has been established of “ruinous or catastrophic” diseases. This definition sought to ensure a specific approach to these diseases, with two aims: to protect the individual by exonerating him or her from the payment of deductibles or co-payments and, the other, to ensure the sustainability of the system, starting from the compulsory payment of contributions for at least 2 years. However, this model has been violated by lawsuits, adverse selection, and other phenomena that have seriously threatened its sustainability. This results in an effective risk to the future protection of patients and, in particular, the lack of alignment of the objectives of insurers and patients: there is no clear incentive in the model to generate an impact on health and improve patient condition. In other health models, such as the case of Peru, for example, there is no model that ensures universal financing for the high cost and treatment of the disease. Indeed, there is only a low level of insurance coverage (61.8 %) with close to 11 million people (38.2 % of the population) unprotected. The disease is considered as part of a fragmented model according to levels of complexity [5, 11].

Additionally, the definition of “ruinous or catastrophic diseases” paradoxically automatically excluded diseases like rheumatoid arthritis, whose health-care costs during a patient’s lifetime well exceed the costs of a coronary angioplasty or other diseases that receive greater attention by the different system agents [1, 5]. The classification of the disease as “ruinous” must therefore take a patient focus. It should mean that payment of sliding scale fees and co-payments should be exempt in diseases such as rheumatoid arthritis, high blood pressure, or asthma. A restriction should be applied to accessing health services where the decision is made by the health-care professional and not by the patient.

The idea behind co-payment is to co-finance the system by the user, and the sliding scale fee seeks to rationalize the use. Given that in RA, services are used frequently and restricting access to such would forms a barrier, the financing of health care must be insured through risk premiums and not in the form of co-financing by patients. The concept must be that no additional charges shall be demanded of the already negligible resources of the insurers but rather the relevant adjustment will be made in the Per Capitation Unit (PCU) ensuring access and attention to those suffering from chronic diseases like RA.

### System fragmentation and deficiency of specialized networks and centers of excellence

The health-care model in Latin American countries is based on the classification of institutions according to levels of complexity (generally classifying from level I to level III or IV, with I being the least complex and III or IV, the most specialized). This typical classification of services fragments the health-care model, ignoring the dynamics that arise in a model that is focused on the patient and their disease [4, 11].

Clearly, we cannot demand that the RA health-care model should have specialized doctors, given that we have already reported on the insufficient number of such and the critical supply/demand ratio in this respect. Indeed, the needs of the major cities can simply not be met, and the supply is simply non-existent throughout much of national territory. The concept of “coordination” implicit in the model described, and which will be detailed further on, can be potentially redeemed.

### Limited supply of services

The lack of appropriate services for the disabled is an important barrier that prevents access to health care. For example, investigations carried out in the states of Uttar Predesh and Tamil Nadu, in India, revealed that after cost, the lack of services was the second most important barrier to the use of medical establishments [7].

### Physical obstacles

Rheumatoid arthritis affects 1 % of the population and is more frequently the cause of disability than heart disease, cancer, or diabetes mellitus. Unequal access to building (hospitals, health centers), an inaccessible medical equipment, poor signage, narrow doors, indoor stairs, inadequate bathrooms, and inaccessible parking areas all create obstacles to the use of health-care structures [7].

The accessibility of health and transport services are two of the main reasons why the disabled simply do not receive the care they need in low-income countries: 32 to 33 % of the non-disabled population cannot afford health care, as compared with 51 to 53 % of disabled people.

### Inadequate skills and knowledge by medical staff

The disabled report a lack of skills among medical staff in attending their needs, twice as often as the non-disabled. They report mistreatment by said staff four times as often and that care is denied to them, three times as often [7].

The aim of early treatment with disease-modifying anti rheumatic drugs (DMARDs) in patients with RA is to suppress the activity of the disease before it causes joint damage. This allows for a better prognosis and potentially results in the remission of the disease for patients who start treatment during the first 3 months. In turn, this results in a lesser risk of disability and incapacity 5 years down the line.

One clear example that reveals the insufficient knowledge about the disease, and thus the ability to identify it in a first consultation and make an early diagnosis, is seen in a study carried out on 98 patients in Mexico in 2010. This found that only 19 % of patients started DMARDs during the first 3 months from when symptoms began. The delay in prescribing DMARDs was caused mainly by the delay in the GP referring the patient to a rheumatologist. Clearly, the late diagnosis of RA, long after symptoms start, is also a contributing factor in the delayed start of treatment with DMARDs. Studies conducted in the last 20 years on the start of treatment with DMARDs in patients with early onset of RA in the United States of America [12], Spain [13], Canada [14], England [15], the Middle East [16], and other European countries [17] show that the average time between the start of symptoms and the start of DMARDs varies between 6 and 18 months, similar to that seen in the Mexican study (average 11 months). Thereby, less than 30 % of RA patients receive treatment with DMARDs during the first 3 months [18–20]. This is why it is important to create strategies that impact the diagnosis of RA and the early start of treatment with DMARDs [21–23].

## Special considerations for Latin America

*There are insufficient qualified people able to treat rheumatoid arthritis*. The World Health Organization (WHO) has established that there must be at least one rheumatology specialist for every 100,000 inhabitants, and this is a long way off current standards. Indeed, in view of the current deficit in specialized human resources, PANLAR has agreed to improve the diagnostic and therapeutic capacity by involving GPs in a critical drive made by society to improve the quality of the population of patients with the disease [17].

*Lack of availability of medications and access to therapies*. Currently recommended RA treatments are very expensive. In Argentina, they can cost up to 89 % of the average lifetime income of the population, and this comes in addition to the problems relating to education, poverty, and lack of health, making access very problematic in this context [17].

*Inadequate information systems and insufficient medical records*. Another means by which to improve the identification of RA is to optimize the systems used to record data in medical records. Effective records containing essential data on epidemiology, standardized forms using CIE-10 codes, demography, employment, absenteeism from work, days of disability, cause of death, and other such information can in fact allow for the early identification, monitoring, and prevention of complications [17].

## Characteristics of a patient-focused health-care model

The characteristics of an ideal health model should be determined according to the needs and resources of the population in which it is to be applied. However, there are always shared objectives to be met, such as the following [11, 24]:

- *Equality* in accessing health care
- *Continuity* of medical attention and treatment
- *Completeness* of the service provided
- *Effectiveness*
- *Joint responsibility* of the medical team, insurer or financing party, and patient and their family.
- *Inter-cultural nature*
- *Empowerment* of the community and patient
- *Social discussion*: that debate on the financing of the disease and the sustainability of the model must be open and the society must make the investments required. What should be included and excluded in and from a benefits plan? Where should investments be made and not be made? Individual ethics vs. collective ethics
- *Humanization*
- *Patient safety*
- *Focus on health results*: patient safety, survival, impact in terms of disability, and disease progression.
- *Satisfaction*: patient expectations must be met. The model must consider the establishment of objectives focused on patient expectations with a view to optimizing the level of reintegration in everyday activities. There must also be an assessment of the degree to which patient expectations are met by the treatment and health-care model. It is not a question of achieving purely medical objectives, nor of satisfying the specialist’s expectations, but rather those of the patient, according to their values, priorities, and the way in which they see life.

## Proposed health-care model

In Colombia (as an example), the population has a health system that covers preventive, curative, and rehabilitation care services. Despite this, however, a series of aspects restrict actual coverage, creating a new reality: the lack of protection of the population due to a failure to align the interests and the model, despite the universal coverage offered by the system.

Today, typically, “gateway” services operate in a capitation method with populations assigned to a given outpatient center. This model effectively restricts access to specialists, whether included in the capitation or brought about with the charge to the insurer following referral. In either case, the model basically allows the gateway to act as a sort of “gate keeper,” restricting access to specialists, partly, of course, to cope with the clear lack of professionals available. In some cases, however, the approach instead stems from the misunderstanding that leaving the patient in the hands of the GP is rational. Yet early access to the specialist, hand-in-hand with risk management assured through a properly trained medical team in primary management, can reduce complications and disease progression. It can also assure a more rational approach to the use of diagnostic methods, less pointless doubling-up of such and pharmacological prescriptions that are more in line with the clinical situation and patient needs.

The proposal includes monitoring a patient according to personal health risk and with a clear aim. The different levels of complexity can be structured and a full, appropriate, specialized care assured, with no restriction to access, no co-payment, no deductible nor sliding scale fees that could potentially restrict access to the services the patient needs and are prescribed by the professional [2, 8].

In this way, the attention paid to high-cost chronic diseases requires a “side flow” model. This therefore establishes the following pillars of the new disease management model:

1. Active search for cases and early identification. This is why resources must be allocated to primary health care to ensure that medical staff and the community are made aware of the signs, symptoms, and criteria for pre-diagnosis. In turn, this results in their empowerment, enabling them to make suitable decisions in requesting qualified services. Thereby, the active search for cases is one of the pillars of the model’s success and takes into account specific actions that aim to have patients seeking consultation during the early stages of the disease, when symptoms are still minor or of recent onset.
  These actions include the following:
  - *Training of the society on signs and symptoms*. The paradigm is who should do it? The government? On what basis? The intervention of each of the players and the sum total of efforts allow us to become a more informed, more empowered society. In this way, we can share examples of individual efforts that generate awareness and a transformation process. In Peru, Oncosalud, a cancer protection institute with more than 700,000 members and 25 years of experience, uses its website and various social networks to permanently send messages about the prevention of the disease. It gives recommendations on healthy lifestyles, how to recognize early signs of the disease, and self-examination techniques, among others. It has even created a contest: PREVENCE, which celebrated its third edition in 2015. This includes a school category with the aim of having Oncosalud process educational digital materials to get the school community involved in developing strategies for disseminating prevention messages, ensuring they “go viral,” rewarding the school and course with the greatest impacted population. The winning school and students obtain a prize in the form of goods and tools to facilitate educational development (tablets, computers, etc.). These prizes are purchased by Oncosalud, which also pays for the costs of the contest, the digital materials and the logistics, and dissemination of materials to the various schools. Once the category was structured, it was presented to the Ministry of Education and the Ministry of Health, making the campaign a part of the curricular mesh developed jointly by the two ministries. Although this strategy will provide initial coverage, it must start empowering the pediatric and general population from an early age. Similar strategies are easily replicated for community education.
  - *Revision of uses*: insurance companies must continue to reinforce a proactive position with regards to the identification of risks. They do so by taking data from their insured parties and implementing processes that revise uses, enabling the identification by consultants of risk signs and symptoms, a more proactive position.
  - *Implementation of* “*Habitual Consultancy*” *programs*: Compensar, in Bogota, Colombia, has implemented processes for monitoring and identifying populations seeking frequent consultations for similar reasons. The most complete analysis of these populations, reasons for consultation, and the directing of these individuals to special, longer consultation makes it possible to define the signs, symptoms, and any risks obtain a precise diagnosis and guide the management of patients showing signs and symptoms of chronic diseases.
2. Insurers need to base care models on the assurance of defined principles. If the network is not accredited, attention should be delegated to an accredited network. This allows insurers to highlight minimum standards for the attention to patients with chronic diseases, in this case, RA patients. These should include the following: training of staff and gateway professionals on RA, specialized support in the form of coaching; availability of specialized staff to provide GPs with support, either by a physical presence or remote medicine; availability of databases enabling the monitoring and identification of the population and the use or care that facilitates the establishment of cohort monitoring processes, optimal medical conditions, safety of installations and equipment, infrastructural conditions enabling all (including disabled) patient access to centers, skilled, trained medical staff in recognizing and managing rheumatic diseases; and extensive knowledge of the route to be taken by the patient, minimizing hindrances and obstacles to save valuable time in affording the patient treatment and encourage him to pursue it. The insurer must have the resources to carry out clinical examinations, provide medications and to teach and train the patient on how to care for their disease. Lastly, the insurer must have an internal entity in charge of regulating due compliance with the national processes and protocols governing the management of RA patients and suitable management of the resources assigned to them.
3. Implementation of the “revision of uses” process: the role of the insurer clearly entails its liability for health care and, therefore, the active search for cases must involve the implementation of a process revising uses with regards to certain parameters (consumption of medications, consultations for certain diagnosis of CIE 10, use or prescription of certain laboratory tests, etc.). This is why access to clinical and paraclinical information is mandatory. The identification of potential cases must be directed towards the network qualified to perform a primary assessment.
4. Adoption of a trans-disciplinary health-care model that runs alongside the gateway capitation model. In this respect, and regardless of the method of payment, any suspected patient should be assessed early on by the trained team (a “case manager”). Thus, when obtaining an approximate diagnosis, the patient will be referred back to the gateway if rejected at diagnosis or will have *coordinated* specialized attention if the diagnosis is confirmed or looks to be highly likely. Starting from this point, the attention process should follow on with specialized centers (treatment providers) in managing or coaching in the cities and municipalities where there is a lack of rheumatology specialists.
5. The implementation of remote medical services granting access to specialists nationwide is essential. By using applications such as e-mail, fax, video conferencing, and WebEx, as well as mobile applications like WhatsApp, Viber, and Skype, along with telephone calls, specialists like the rheumatologist, radiologist, cardiologist, and physiatrist can be consulted in primary or secondary attention centers. The doctor therefore obtains more information helping them make decisions, and greater skills, effectively benefiting both the doctor and patient alike. Moreover, in this model, remote medicine becomes an essential tool for the patient. Indeed, it enables constant, direct communication with the doctor or medical staff, thereby settling doubts, reinforcing healthy behavior and improving adherence to treatment with no need to move from their home or place of work.
6. The human resource medical training policy needs to be revised, assigning resources to increase the availability of specialties with a careful analysis of supply and demand according to territory, region, and city.
7. Redefinition of the benefits plan. The incorporation of medical technologies, extensively discussed in terms of the sustainability of the social security systems, must also undergo major changes. There must be an incorporation of drugs and technology to satisfy the solid evidence with clear cost-effectiveness, cost-benefits, cost-minimization studies, among others, but the process must be defined and coupled in such a way as to flow effectively. Prescription under the protective figure, CTC, protective or other legal proceedings, significantly reduces the possibility of implementing clinical practice guides and disease management. The disease should therefore clearly be managed strictly in accordance with clinical practice guides and the therapeutic lines and scales defined therein.
8. The definition of a disease as being “ruinous” or “catastrophic” must include entries such as rheumatoid arthritis, meaning that the non-application of co-payments and sliding scale fees can successfully eliminate the access barrier that naturally follows in respect of a disease whose prescriptions, procedures, and interventions are to a large extent carried out in outpatient clinics, with the patient paying for each of the services. The concept of “sliding scale fees,” aiming to moderate use of services decided by the patient, is a more complex matter. It would often, in fact, appear to be rather contradictory given that patient adherence to treatment is required, their use of the preventive and rehabilitation services, yet the application of sliding scale fees to services means that the patient, rather than the doctor, may say NO to such. In this way, the concept to be applied would perhaps be more appropriate as “co-payment,” as a co-financing element, yet as already explained, this must be exonerated and financing should be involved in a first adjustment for risk following a careful analysis so as to correct the effect on the system's sustainability.
9. There must be a system to measure the process of attention and assessment of management according to outcomes by each of the insurers and/or qualified networks to provide the services, incorporating indicators with a patient focus. This will result in an about-turn in the definition of “patient journey” and the care process.
10. Regarding the previous point, it is also essential to assess patient satisfaction with the level of resumption of their day-to-day activities. The key objective of the model should be to maintain and preserve patient satisfaction with their level of recovery, classified as the degree to which they can resume their everyday activities. What is truly important in a health-care model adopted to deal with chronic diseases is the quality of life achieved. It is not a question of adding “years to life,” but rather “life to years.”
11. In the stages into which medical science breaks down the process of recovery and the disease, which unfortunately evolves towards a fatal outcome and clearly limited function, the health system and attention process must go hand-in-hand with home assistance. This home assistance should include pain management, infection management, support with cardiovascular deficiencies, among others, as well as ensuring the basic training of caregivers. Said caregivers may be family members or persons appointed by them, but in any case, it is clear that the family takes primary responsibility for caring for their loved ones and this basic, social, nutritional, and living care cannot be delegated, as the health system is only responsible for medical treatments. We cannot allow for a model where the families “abandon” patients to the mercy of the health system, even if still at home. And, therefore, the dissemination of a model of the “rights and *duties*” of the users and caregivers, as a condition for the patient’s admission to the disease management model, must involve the signing and specific acceptance by these persons, even potentially considering the application of “pedagogic sanctions.”
12. Implementation of means intended to guarantee *patient safety* throughout the health-care period [25]:
  The standardization of a system seeking out risk factors, failings, and adverse events.
  The suitable reporting of adverse events so that corrective action can be taken to avoid new cases.
  The investigation, analysis, management, and decision-making to avoid foreseeable adverse events and, if such should arise, to mitigate their consequences.
  The organization defines if the current care is the consequence of an adverse event, regardless of where the previous care was given.
  From access, redundant identification mechanisms of the patient are defined.
  From access, the risks of care are identified, according to the type of user. Said risks should be recorded in a specific section of the medical records, where all those involved in caring for the patient can be warned to avoid adverse events during their care.
13. The gateway of primary care is converted in this model into the body that, as part of the care flow, is responsible for initiating the other strategies, by which, as described previously, the GP shall have the information available to him to allow him to feel sure about taking decisions with the patient seeking their consultation. This information may be supplied by: remote medicine applications enabling him to communicate with the rheumatologist and other specialist doctors who can provide real-time guidance on diagnosis and treatment if there are any doubts; teleconferencing and video conferencing (again provided by specialists), such as academic discussions involving current clinical practice guidelines regarding the population with which they works. They shall also be granted access to RA treatment protocols supplied by the insurer when the doctor is contacted and thereafter assessed in day-to-day practice in terms of their correct application. All this facilitates good clinical practice, reduces the probability of unnecessary referrals to specialists, thereby saving time and resources.
14. When taking the patient into consideration, the places where they or she receives medical attention must be adjusted to overcome any accessibility obstacles and ensure greater adherence to treatment. This is why in caring for RA patients, *health-care centers or clinics must have the following characteristics*: wide access doors to allow wheelchairs in and the health-care center must have sufficient chairs for patients and their caregivers and potentially several members of family who may attend the visit in a comfortable manner. Ideally, there should also be a place to perform case histories, which would be more like a visiting room and have no barrier in the form of a large desk separating the doctor from their patient. The center should also have material to enable the doctor to graphically explain the disease to the patient and make sure their recommendations are fully understood. The health-care center should also have the following:
  An examination area
  Area separation barriers
  Medical unit
  Sinks
  Natural or artificial ventilation
  Natural or artificial lighting

The center should have the following equipment: scale, blood pressure monitor, height rod, stethoscope, meter ruler, hearing equipment, goniometer, x-ray viewer, treatment trolley, lamp, stretcher, stepladder (with two steps), desk, chairs, screen with two parts, filing cabinet, hospital waste containers with lid, and sharps containers.

All these are basic requirements to guarantee suitable patient care.
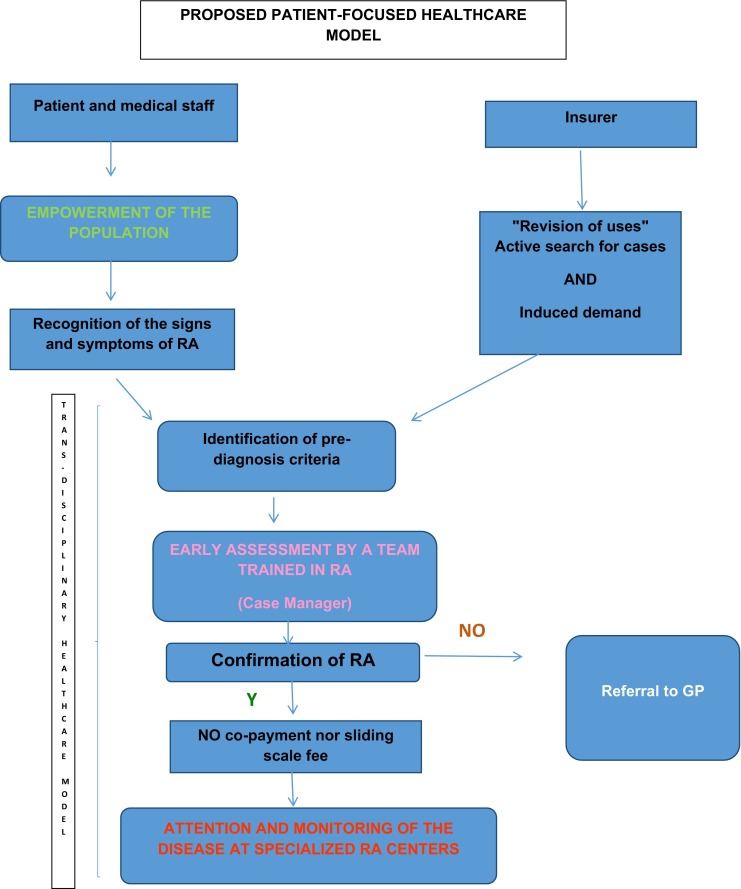


## Elements necessary for handling RA as a chronic medical condition

To develop the structures and processes that enable rheumatoid arthritis (RA) patients to reach stable conditions, to improve or even go into remission of symptoms, as applicable, and to keep the patient in these conditions [1, 8, 18, 20], in these cases, the following elements are particularly critical:

1. Education and support in self-care, particularly in the community, at work, and in social settings in general. As part of its promotion and prevention programs, each insurer shall configure this model as an essential part of patient education, empowering the patient with respect to their disease. Once the patient has been diagnosed with rheumatoid arthritis, they will automatically be included in their insurer’s *school of patients*. Here, talks will be held by an interdisciplinary group of medical professions (such as doctors, physiotherapists, nutritionists, psychologists, and nurses) and by other patients acting as positive examples in disease management, on RA-related matters. These will generate support in changing habits and adopting healthy lifestyles, such as changes in working routines, dietary changes, and the implementation of joint protection techniques. Virtual care (by means of video conferencing) or physical attendance of such activities must be compulsory for diagnosed patients. It shall therefore be a requirement for continuing the program, receiving medication and benefiting from the exemption from co-payment of care.
2. Support in changing habits and adopting healthy lifestyles, as well as making changes to working routines, dietary changes, and the implementation of joint protection techniques.
3. Support and monitoring of adherence to pharmacological and non-pharmacological therapy.
4. Patient contact with their personal doctor of the *center specialized in RA* (*treatment provider*) in the event of worsening or acute episodes.
5. Patient contact with their treatment provider to solve minor day-to-day needs, using different contact methods such as telephone, e-mail, text messages, chat, web and videos.
6. Implementation of remote medicine channels. For cities or municipalities without rheumatology specialists, an area must be set up with trained human resources. This can therefore act as a specialized RA care center for these populations, and the professionals will be tutored by the rheumatologists and other specialists leading the program by means of IT and communication tools [19].
7. To ensure the evaluation of the patient by rheumatology, physiotherapy, and occupational therapy immediately following confirmation of a diagnosis of RA and according to the patient's individual needs as recorded by their treatment provider.

To use clinical management tools that successfully optimize use of resources according to clinical results and quality of life, such as:

1. Development and adoption of protocols for highly standardized processes and verification of strict application.
2. Development, adoption, and bringing up-to-date of clinical practice guides for less standardized processes, verifying their application and regularly analyzing possible deviations.
3. Detailed monitoring of the indicators of clinical results and quality of life and of the ways resources are used and the relevant impact on the provider’s prospective budget.
4. Individual patient monitoring by a team with extensive knowledge of the disease, the available resources, processes and objectives.
5. Implementation of improvement cycles in order to obtain better clinical and economic results.
6. Unification of the medical history to keep all patient and disease data together.
7. Medical committee or council to define biological therapy [26].

To develop IT tools that can monitor internal processes, patient communication with their provider and support remote medicine communication channels between specialized and non-specialized doctors, physiotherapists, nurses, and other members of the team. These can run between different specialized care centers to guide patient management [26, 27].

## Referral and counter-referral of RA patients

To achieve a suitable RA patient journey and optimize resources, this model suggests that the RA patient should be assessed by the specialist in: rheumatology, physiatry, and occupational health, always in the following two cases:

1. Once the RA has been diagnosed on a first level or at gateway, in compliance with the clinical and paraclinical criteria assessed by the doctor according to the protocols and clinical guides adopted by the Ministry of Health for application throughout the national territory.
2. If the patient has any complications or clinical situations that have not been successfully solved, despite the use of all resources available by the first level treatment provider, and which have proved to be insufficient, despite good adherence by the patient to treatment, due to the complexity of the case.

Referral to other supporting professionals (psychologist, social worker, nutritionist, nurse) will be made according to the specific needs of each patient and as considered relevant by the primary physician, at any time.

The rest of the consultations for optimizing and monitoring RA patients will be organized by the primary physician, the multidisciplinary team trained to manage these types of patients. Tangible benefits will ensue for both the patient and the health system, as patients living in rural areas can be avoided the costs and inconveniences involved in moving to 3rd or 4th level centers for specialized medical consults. Indeed, these roles are replaced by the nearby GP (who knows the patient and their family) and other professionals trained by the closest primary medical center (treatment provider) to manage this type of pathology. These professionals shall act under the direction of a chief rheumatologist who will make remote contact using the remote medicine applications explained, avoiding months of waiting to see the specialist, as a result of the severe lack of such and the great congestion of the health system, which also affects adherence and continuity of treatment and patient monitoring. It regulates the costs of the health system, ensuring fewer disease-related complications, and avoiding mass affluence of patients to the clinics and hospitals of greater complexity [20, 26, 28].
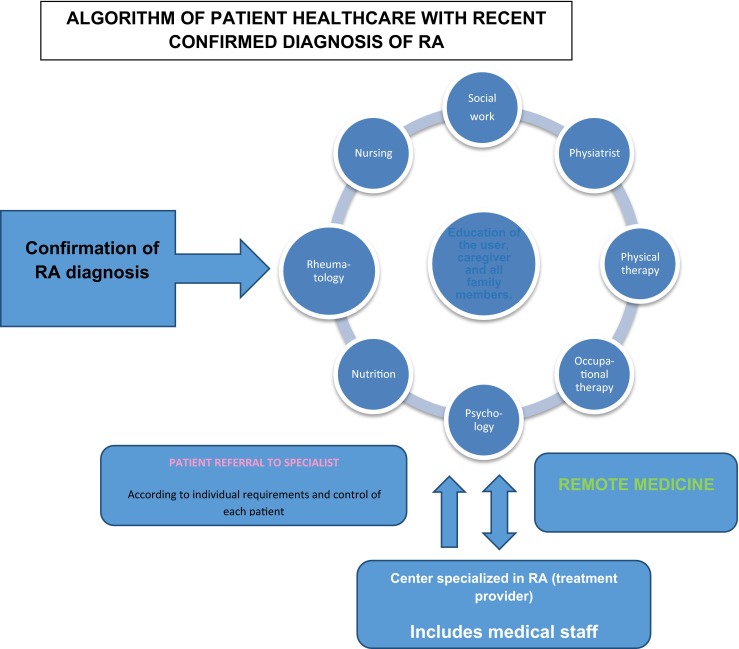

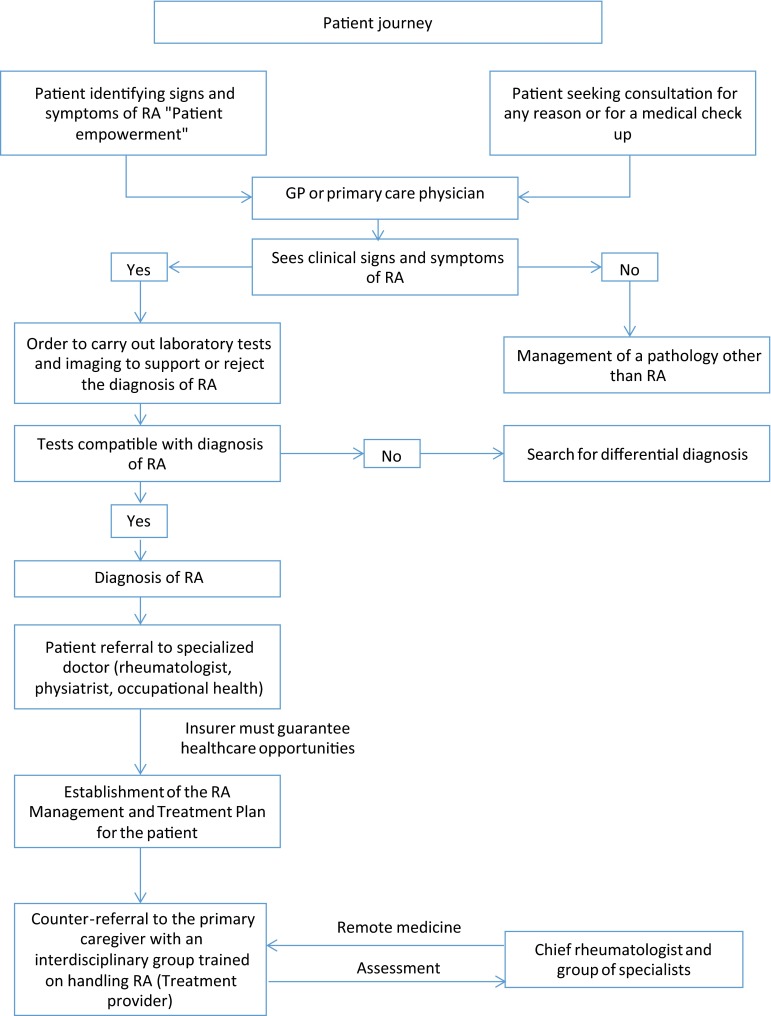


## Indicators to mediate the results

### Remission

Definition: Percentage of people diagnosed with RA and who are in remission after 12 months of monitoring. Remission will be defined according to EULAR criteria.
Calculation: ᅟ
Denominator: Number of patients who have received a diagnosis of RA.
Numerator: Part of the denominator in remission.

### Disability

Definition: Percentage of people diagnosed with RA who, as a result of the disease, suffer some form of limitation to joint function.
Calculation: ᅟ
Denominator: Number of patients who have received a diagnosis of RA.
Numerator: Number of patients suffering from some form of limitation to joint function caused by the disease (number of anatomical regions with severe or limiting reduced function) or number of days of working disability or of days of functional disability.

### Use of orthopedic surgery

Definition: Percentage of patients diagnosed with RA who have had surgery as compared with total number of cases monitored.
Calculation: ᅟ
Denominator: Patients who have received a diagnosis of RA during monitoring.
Numerator: Part of the denominator that has received orthopedic surgery.

## Discussion

### Are we ready?

The challenges faced by the systems in Latin America have three separate dimensions: financing, service supply, and quality of services. Each of these spheres must be developed in a harmonious, balanced manner. Progress has been made in various countries with regards to financing, with combined public and private efforts made, yet major contributions are still required to ensure universal coverage. As concerns quality of services, there has been growing interest in health certification and accreditation processes, inspection, auditing, and control models, accreditation processes, patient safety models, and other elements involved in the disease management. Clearly, these are now starting to draw a line towards a permanent route in the search for quality. Finally, the development of the treatment infrastructure requires not only the construction of the physical infrastructure but also the training of the human resources. In the countries of our region, this matter requires some form of effort made by the states, private enterprise, the world of academia, and the other players involved in the health systems, so as to ensure that the population can access specialized professionals in different areas of our territories. These new professionals must ensure the provision of services based on clear, solid principles of rationality and relevance that can, over time, facilitate the financial sustainability of the model. The structure with trained health professional networks and primary health-care models with health coaching, the monitoring of cohorts, and other forms of managed care mechanisms, take more effort than technology.

Finally, a change is required in the collective conscience and an in-depth social debate that can prioritize and focus resources, solving the conflict of individual ethics vs. collective ethics. Complete well-being cannot be assured, yet we can work towards building a better society.
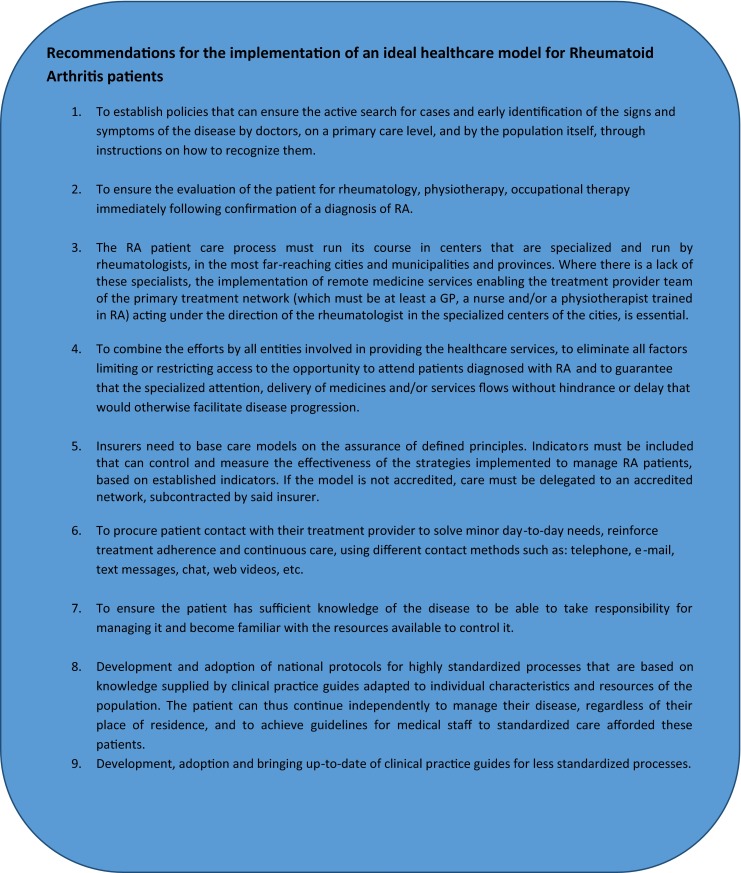


## References

[1] Emery P, Breedveld FC, Dougados M, Kalden JR, Schiff MH, Smolen JS (2002). Early referral recommendation for newly diagnosed rheumatoid arthritis: evidence based development of a clinical guide. Ann Rheum Dis.

[2] Solomon DH, Bates DW, Panush RS, Katz JN (1997). Costs, outcomes, and patient satisfaction by provider type for patients with rheumatic and musculoskeletal conditions: a critical review of the literature and proposed methodologic standards. Ann Intern Med.

[3] Ambriz Murillo Y, Menor Almagro R, Campos-González ID, Cardiel MH (2015). Health related quality of life in rheumatoid arthritis, osteoarthritis, diabetes mellitus, end stage renal disease and geriatric subjects. Experience from a general hospital in Mexico. Reumatol Clin.

[4] Daniel Roig V, Montserrat Nuñez Juárez B, Esther Nuñez Juárez C, José Luis Del Val García C, Alex Sánchez P, Maria Bonet L, En nombre del grupo ARQUALIS1 (2009). Characterization of patients with rheumatoid arthritis according to the health care level. Reumatol Clin.

[5] Carlo Vinicio Caballero U (2004). Artritis reumatoide como enfermedad de alto costo. Rev Colomb Reumatol.

[6] Vol. 11 – Núm. 2 – Febrero 2001 MEDIFAM 2001; 11: 47-54 ARTÍCULO ESPECIAL Gestión de Enfermedades (Disease Management). Una aproximación integral a la provisión de cuidados sanitarios

[7] Mustafa Al M, Femi A, Jamal Al S, Yousef Al W, Gerd-Rüdiger B, Maurizio C, Joseph F, Lyn M, Heather MD-B, Kevin P, Carlos P, Carter T, Kvien TK (2015). The global challenges and opportunities in the practice of rheumatology: white paper by the world forum on rheumatic and musculoskeletal diseases. Clin Rheumatol.

[8] Skouen JA, Grasdal A, Haldorsen EMH (2006). Return to work after comparing outpatient patient multidisciplinary treatment programs versus treatment in general practice for patients with chronic widespread pain. Eur J Pain.

[9] Cardiel MH, Díaz-Borjón A, del Mercado Espinosa MV, Gámez-Nava JI, Barile Fabris LA, Pacheco Tena C, Silveira Torre LH, Pascual Ramos V, Goycochea Robles MV, Aguilar Arreola JE, González Díaz V, Alvarez Nemegyei J, del González-López LC, Salazar Áramo M, Portela Hernández M, Castro Colín Z, Xibillé Friedman DX, Alvarez Hernández E, Casasola Vargas J, Cortés Hernández M, Flores-Alvarado DE, Martínez Martínez LA, Vega-Morales D, Flores-Suárez LF, Medrano Ramírez G, Barrera Cruz A, García González A, López López SM, Rosete Reyes A, Espinosa Morales R (2014). Update of the Mexican College of Rheumatology guidelines for the pharmacologic treatment of rheumatoid arthritis. Reumatol Clin.

[10] Álvarez-Hernández E, Peláez-Ballestas I, Boonen A, Vázquez-Mellado J, Hernández-Garduño A, Rivera FC, Teran-Estrada L, Ventura-Ríos L, Ramos-Remus C, Skinner-Taylor C, Goycochea-Robles MV, Bernard-Medina AG, Burgos-Vargas R (2012). Catastrophic health expenses and impoverishment of households of patients with rheumatoid arthritis. Reumatol Clin.

[11] Stoffer MA, Smolen JS, Anthony W, Ales A, Ailsa B, Loreto C, Veronika F-M, Estibaliz L, Pawel O, Petersson IF, Till U, Stamm TA, the eumusc.net-working group (2014). Development of patient-centred standards of care for rheumatoid arthritis in Europe: the eumusc.net project. Ann Rheum Dis.

[12] Chan A, Felson TD, Yood AR, Walker MA (1994). The lag time between onset of symptoms and diagnosis of rheumatoid arthritis. Arthritis Rheum.

[13] Hernández-García C, Vargas E, Abásolo L, Lajas C, Bellajdell B, Morado IC (2000). Lag time between onset of symptoms and access to rheumatology care and DMARD therapy in a cohort of patients with rheumatoid arthritis. J Rheumatol.

[14] Jamal S, Alibhai SM, Badley EM, Bombardier C (2011). Time to treatment for new patients with rheumatoid arthritis in a major metropolitan city. J Rheumatol.

[15] Kumar K, Daley E, Carruthers DM, Situnayake D, Gordon C, Grindulis K (2007). Delay in presentation to primary care physicians is the main reason why patients with rheumatoid arthritis are seen late by rheumatologists. Rheumatology (Oxford).

[16] Zafar S, Badsha H, Mofti A, Delosantos A, Altares J, Matudio G (2012). Efforts to increase public awareness may result in more timely diagnosis of rheumatoid arthritis. J Clin Rheumatol.

[17] Raza K, Stack R, Kumar K, Filer A, Detert J, Bastian H (2011). Delays in assessment of patients with rheumatoid arthritis: variations across Europe. Ann Rheum Dis.

[18] Muñoz Alamo M, Ruiz Moral R, Pérula de Torres LA (2002). Evaluation of a patient centred approach in generalized musculoskeletal chronic pain/fibromyalgia patients in primary care. Patient Educ Couns.

[19] Machado P, Castrejon I, Katchamart W, Koevoets R, Kuriya B, Schoels M (2011). Multinational evidence-based recommendations on how to investigate and follow-up undifferentiated peripheral inflammatory arthritis: integrating systematic literature research and expert opinion of a broad international panel of rheumatologists in the 3E Initiative. Ann Rheum Dis.

[20] Smolen JS, Daniel A, Bijlsma JWJ (2010). Treating rheumatoid arthritis to target: recommendations of an international task force. Ann Rheum Dis.

[21] Van der Heide A, Jacobs JW, Biglsma WA, Heurkens AH, van Booma-Frankfort C, van der Veen MJ (1996). The effectiveness of early treatment with second line antirreheumatic drugs: a randomised controlled trial. Ann Intern Med.

[22] Van der Linden MP, le Cessie S, Raza K, van der Woude D, Knevel R, Huizinga TW (2010). Long-term impact of delay in assessment of patients with early arthritis. Arthritis Rheum.

[23] Mottonen R, Hammonnen P, Leirisalo- Rapo M, Nissila M, Kauntiamen H, Korpela M (1997). Comparison of combination therapy with single-drug therapy in early rheumatoid arthritis: a randomised trial. Lancet.

[24] Romero RV, Ramírez NA, Méndez PAM, Vélez OR (2009) Atención Primaria Integral de Salud, Estrategia para la Transformación del Sistema de Salud y el Logro de la Equidad en Salud, 1ra edición ISBN 978-958-8545-01-1 Bogotá D.C, Colombia

[25] Manual de Acreditación en Salud Ambulatorio y Hospitalario Colombia (2011) ; Versión 003 Descriptores Acreditación—Accreditation Dirección General de Calidad de Servicios Unidad Sectorial de Normalización en Salud. Ministerio de la Protección Social - ISBN: 978-958-8717-33-3. Bogotá D.C, Colombia

[26] Smolen JS, Robert L, Breedveld FC (2013). EULAR recommendations for the management of rheumatoid arthritis with synthetic and biological disease-modifying antirheumatic drugs: 2013 update. Ann Rheum Dis.

[27] Actualización de la guia de practica clinica para el manejo de la artritis reumatoide en España, GUIPCAR2007, Soc Esp Reumatol

[28] Latin American Rheumatology Associations of the Pan-American League of Associations for Rheumatology (PANLAR) and the Grupo Latinoamericano de Estudio de Artritis Reumatoide (GLADAR) (2006). First Latin American position paper on the pharmacological treatment of rheumatoid arthritis. Rheumatology.

